# Have I Been Touched? Subjective and Objective Aspects of Tactile Awareness

**DOI:** 10.3390/brainsci14070653

**Published:** 2024-06-27

**Authors:** Emanuele Cirillo, Claudio Zavattaro, Roberto Gammeri, Hilary Serra, Raffaella Ricci, Anna Berti

**Affiliations:** Department of Psychology, University of Turin, Via Verdi 10, 10124 Turin, Italy; emanuele.cirillo@unito.it (E.C.); claudio.zavattaro@unito.it (C.Z.); roberto.gammeri@unito.it (R.G.); hilary.serra@unito.it (H.S.); raffaella.ricci@unito.it (R.R.)

**Keywords:** tactile awareness, tactile consciousness, tactile perception, touch sensation, rubber hand illusion, mirror box illusion, somatoparaphrenia, embodiment, body integrity disphoria

## Abstract

Somatosensory tactile experience is a key aspect of our interaction with the environment. It is involved in object manipulation, in the planning and control of actions and, in its affective components, in the relationships with other individuals. It is also a foundational component of body awareness. An intriguing aspect of sensory perception in general and tactile perception in particular is the way in which stimulation comes to consciousness. Indeed, although being aware of something seems a rather self-evident and monolithic aspect of our mental states, sensory awareness may be in fact modulated by many different processes that impact on the mere stimulation of the skin, including the way in which we perceive our bodies as belonging to us. In this review, we first took into consideration the pathological conditions of absence of phenomenal experience of touch, in the presence of implicit processing, as initial models for understanding the neural bases of conscious tactile experience. Subsequently, we discussed cases of tactile illusions both in normal subjects and in brain-damaged patients which help to understand which high-order processes impact tactile awareness. Finally, we discussed the observations reported in the review in light of some influential models of touch and body representation.

## 1. Introduction

Touch, a fundamental component of the somatosensory system, is always present in our experience and, therefore, in our mental life and is a stimulation that continuously affects our body, interacting with other senses and especially with the feeling of body ownership [1]. Tactile processing and in particular tactile awareness, together with conscious experience in other modalities, is therefore fundamental for the construction of a coherent sense of self [2]. The sense of touch has been traditionally considered part of the somatosensory system which, along with the other senses, collects input that informs us about the external environment and our internal states. The somatosensory system consists of an intricate and complex set of receptors and nerve pathways that detect and process any mechanical stimulation that affects our bodies [3]. Amongst these stimulations, signals that inform about the state of muscles and joints allow the perception of body part position with respect to other body parts and space (proprioception, [4]), while signals coming from internal organs inform about sensations coming from inside of our body (interoception, [5]). Both these components of the somatosensory system collect information from receptors that are deeply localized in our body. Touch (including pain and thermal perception) differs somehow from proprioception and interoception because it depends on more superficial receptors located under the skin, which is the structure that separates us from the outside world, bounding the body space and allowing us to feel contact with other individuals and objects around us. In particular, different receptors like Merkel’s cells, Pacinian, Meissner’s and Ruffini’s corpuscles transmit information about the pressure, duration, and location of a tactile sensation, converting physical stimuli into electrical signals through sensory transduction, generating receptor potentials that may trigger action potentials in primary afferent neurons [3,6]. These neurons transmit sensory information to the central nervous system. They cross to the contralateral side before ascending through the medial lemniscus to the ventrolateral posterior (VLP) and ventromedial posterior (VMP) nuclei of the thalamus and from these nuclei to the primary somatosensory cortex (S1), located in the post-central cortex, and to the secondary somatosensory cortex (S2) located more caudally in the parietal lobe [6]. Interestingly, the ventral and the lateral posterior thalamic nuclei also project to premotor and posterior parietal cortices. These pathways (in Serino and Haggard’s [1] model indicated as pathway 1; see also [7]) reach the dedicated cortex via the subcortical structure in a manner analogous to other sensory modalities, particularly vision.

The sense of touch, however, does not operate in isolation, as it constantly interacts with other senses to form a coherent and cohesive sensory experience. Indeed, while according to the traditional view the different sensory signals are processed in anatomically segregated regions by primary sensory cortices and then combined in higher-order multisensory areas, in recent years, it has been shown that modality-specific brain regions can be affected by multisensory signals. A number of studies have shown how the response to unimodal stimuli in unisensory cortices can be modulated by concurrent presentation of stimuli from other modalities (i.e., multisensory stimulation, see [8,9,10]. From a behavioral point of view, an example of multisensory interaction is that of the visual enhancement of touch (VET) effect [11], where seeing a body part being touched can improve tactile detection on that body part. Another example of how a different sensory modality can affect tactile perception is the effect that the vestibular system has on somatosensory perception, where a reduction of vestibular input reduces the felt intensity of a tactile stimulus [12]. From a physiological point of view, there is evidence that primary sensory cortices can be activated by stimuli presented in a different modality with respect to the one coded by that cortex. Keuhn et al. [13] showed that in area 3b of the human primary somatosensory cortex, fine-grained finger maps are activated not only during tactile mechanical stimulation but also when viewing the same fingers being touched (the authors called this interaction effect ‘foreign sources’ stimulation). Finally, multisensory integration also underlies the body illusions that will be discussed later in this paper. By interacting with other senses, tactile processing enables us to cope with the environment, signaling contact with objects in space, and properly recognize (when vision is obstructed or prevented) the objects toward which our voluntary actions are directed (haptic object recognition) [14]. Moreover, touch sensation is fundamental to perceiving the spatial limits and metrics of personal and peripersonal space (e.g., [15,16,17,18,19]), playing a key role in the recognition of our body as belonging to us, thus distinguishing it from others’ bodies (body ownership, see below and, e.g., [20,21,22,23]). Related to the perception of one’s own body and the bodies of others, touch can also assume an affective meaning that can convey to people a sense of pleasure and closeness (affective touch [24]). As pointed out by De Haan and Dijkerman [25] in their seminal paper on touch circuits, recent studies suggest that different, somehow distributed, and, most importantly, partially overlapping networks are involved in touch perception based on the specific task to be accomplished. This implies that the network underlying both primary processing and more cognitive tasks is less segregated than originally suggested. As a consequence, the model proposed by De Haan and Dijkerman took into account all possible components and interactions between the circuits where tactile stimuli are in different ways involved. In this paper, instead, we considered how awareness of a simple tactile stimulus given on the skin is constructed, focusing both on the subjective experience of touch when a stimulus is applied or impacts on the subject’s body and on the objective data that can verify the subject’s report. Indeed, one of the most intriguing questions posed by neuroscience is how external stimuli come to be experienced as conscious and, therefore, what mechanisms and brain circuits are responsible for sensory awareness [7,26]. It is therefore important to clarify that two aspects of consciousness will be considered here. The first is based on subjective phenomenal experience (first-person perspective), reported by the subject upon the occurrence of certain events. It has long been debated whether subjective reports can be accepted as data in scientific discourse, since the introspection that generates them is often considered aleatory and not objectively verifiable. However, many authors have criticized this prejudice because, while recognizing the methodological difficulty of considering the experience reported by the subjects, they can open an important window into the mechanisms of consciousness [27,28,29,30,31]. The second is based on objective data (third-person perspective) about the conscious experience of touch, to rule out the possibility of subjective reports being the result of mere confabulation. In other words, in this review, we will assess how subjective reports related to the phenomenal experience of touch can be a starting point for understanding tactile awareness and how they can lead to the formulation of hypotheses that can be objectively tested by behavioral experiments or by connecting the subjects’ phenomenal experiences to physiological responses and anatomical data. Thus, the question posed by this review is whether it is possible with the current state of our knowledge to indicate the physiological conditions and neural basis of tactile consciousness.

In everyday life, the subjective experience of touch is usually perceived as directly contingent on the external events that generated it because of the usual correspondence between the actual presence (or absence) of a stimulus and the feeling (or absence of feeling) of being touched. There are, however, counterintuitive conditions where the phenomenal experience does not correspond to the actual stimulation. To this respect, we shall first review clinical conditions in which the processing of stimuli that do not enter consciousness can nonetheless guide patient’s behavior in the attempt to establish whether implicit and explicit processing of stimuli may follow different anatomophysiological pathways. Then, we shall discuss some abnormal phenomenal experiences of touch in the absence of neurological damage and illusory experiences of touch in brain-damaged patients where subjects may feel a touch on their body when in fact no stimulation has been given and, conversely, have no tactile experience despite the presence of stimulation. With reference to the correspondence between stimulation and tactile experience reported by subjects in the different experiments, we will define as ‘veridical’ the condition in which the subjective report corresponds to the presence/absence of a sensory event and ‘non-veridical’ when it does not. Comparison of veridical and non-veridical touch perceptions and the conditions that determine them can unveil what processes, in addition to primary sensory analyses, modulate touch parameters regardless of the existence of true stimulation. This may allow us to disclose the necessary and sufficient conditions for tactile awareness to be generated under different circumstances. It must also be kept in mind that, as mentioned above, touch and more generally somatosensory experiences have a special feature that other sensations do not have, which is the close relationship to body perception. Indeed, there is a two-way relationship between the subjective experience of touch (the feeling that a touch stimulus is given to my body) and the feeling of body ownership (i.e., the feeling that this body belongs to me). In the words of Serino and Haggard, ‘the receptor organ for touch, the skin, also forms the surface of the physical body’ [1]. Consequently, situations in both normal subjects and brain-injured patients, where the close bottom-up and top-down relationships between touch and body affect one another, will also be considered. With this respect, we will refer to models of touch proposed by Gallace and Spence [7,26] and models of body representation proposed by Tsakiris and Haggard [23] and Serino and Haggard [1]; these which involve both top-down and bottom-up processes in the construction of body representation. We will also refer to a revision of these models recently proposed by us [32]. This part of the review aims to show how the awareness of a simple touch is not simply anchored to the receptors on the skin that have been activated by the stimulation but also to how we experience our body.

## 2. Processing without Awareness in the Tactile Domain

One of the most effective ways of understanding what mechanisms underlie conscious processing is to study those neuropsychological syndromes where it is possible to demonstrate stimulus processing without awareness. That is, syndromes where although analysis of environmental events does occur, that analysis does not produce a conscious experience of those events. These situations make it possible to investigate what kind of processing and what brain circuits are necessary and, if any, sufficient, to have a conscious experience of the external stimuli. Although Gallace and Spence [7,26] in their seminal papers have emphasized the differences between the tactile and visual domains in information processing, we want instead to examine the similarities shared by the two systems in order to find common mechanisms for the emergence of conscious experience (see Table 1). When we talk about common mechanisms, we are referring to the modes of processing information, not the pathways related to the two systems that are obviously segregated, especially in the early stages of transmission and processing of input data. The similarities between the mechanisms of conscious elaboration of touch and vision can be found for both the primary processing of incoming stimuli (as suggested by the blindsight and blindtouch conditions) and for the control systems that impact the conscious processing of stimuli when stimuli that could be normally processed because the primary cortex devoted to their elaboration is intact are in fact not detected consciously (as in the extinction phenomenon, see [33,34]). These conditions will therefore be described in detail in the following paragraphs. Because there is much more evidence in the visual system than in the tactile system, data on the visual system that can inform research on conscious tactile processing and may help in developing hypotheses related to conscious touch perception are presented first (for the similarities between conscious/non-conscious experience in vision and touch, see Table 1).

In neuroscience and neurology, one of the earliest cases of processing without awareness was that of blindsight, where it was shown that visual stimuli can be processed either in a complete absence of visual experience (type 1 blindsight), or with a subjective feeling of experience without precise sensory connotation (type 2 blindsight) (see below). Many studies have been conducted over the years that have addressed behavioral as well as anatomical and physiological questions of the mechanisms involved in the phenomenon of the absence of conscious vision in the presence of stimulus processing (for a recent review see [35,36]). Blindsight is, therefore, a good reference model both clinically and theoretically. Despite the diversity and peculiarities inherent in each sensory modality, it is reasonable to think that sensory experience is constructed through similar, although domain-specific, mechanisms. We will therefore briefly recall here some aspects of blindsight that seem to us to be crucial for the understanding of the phenomenal experience of sensory events in general and of tactile events in particular.

First described in the mid-1970s by Pöppel and colleagues [37] and Weiskrantz and colleagues [38], blindsight is a condition where individuals, after damage to the primary visual cortex that causes blindness in the opposite visual field, can respond to certain aspects of visual stimuli presented in the blind field without having any actual phenomenal experience of them. For instance, if forced to make a guess on the location or direction of a visual event, blindsight patients are able to point toward the stimulus location or to correctly indicate in which direction the stimulus moves, despite claiming that they have not seen anything [39]. The presence of correct responses in the absence of phenomenal experience, in addition to undermining some commonsense idea on consciousness (e.g., ‘If I do not see something I cannot act on it’, see [40]), raises two main questions. First, what are the mechanisms responsible for full awareness? Second, what are the mechanisms underlying processing without awareness? The possibility that consciousness is a threshold phenomenon that occurs when there is a sufficient amount of neural activation would predict the presence in blindsight subjects of residual brain tissue in V1 that would ensure enough activity for implicit but not explicit awareness. This hypothesis was, however, discarded because the blindsight phenomenon can be observed even in the total absence of cortical tissue, as in the case of hemispherectomies [41]. An alternative hypothesis is that there may be dedicated pathways for explicit and implicit aspects of consciousness, suggesting for blindsight the existence of alternative visual routes outside V1 that enable processing of visual stimuli (or at least of some of its prerogatives) even in the absence of conscious perception. Weiskrantz and coworkers [42] found that it was possible in the same patient, by modulating certain stimulus parameters, to switch from type 1 blind-sight, characterized by a total absence of visual experience, to type 2 blindsight, where patients report feeling that something has happened in the visual field. This observation inspired the study of brain activities in the different ‘aware’ and ‘unaware’ modes, which showed a shift in the pattern of activity from the neocortex in the aware mode to subcortical structures in the unaware mode. In particular, it was found that in the modes characterized by some form of awareness (type 2 blindsight and normal vision of the opposite hemifield), peristriate and prefrontal areas (in particular area 46) were activated, while in the unaware modes, the main structure involved was the superior colliculus [43]. These results were later confirmed in a study by Tamietto and colleagues [44]. The anatomical results, especially the activation of the prefrontal cortex in the aware mode, led many authors to claim that V1 may be necessary but not sufficient for explicit knowledge of visual sensory events. The blindsight phenomenon, in addition to indicating the possibility of processing without awareness, is also an example of false belief (the claim of not seeing anything despite the ability to respond to some characteristics of the stimulus) not due to a mere verbal confabulation but to the activation of specific brain areas dedicated to implicit but not explicit processing.

Regarding the sense of touch, Paillard and coworkers [45] reported the first case of dissociation between explicit and implicit touch processing in a patient who, following a left hemisphere stroke that had produced parietal lobe ischemia, had developed complete anesthesia of the contralateral body. Despite the complete inability to feel tactile stimuli even after consistent pressure on the skin, the patient was able to localize stimuli that she did not experience. This symptomatology resembles that of blindsight, and in fact, the authors suggested the term ‘blindtouch’. Even the patient’s comments were similar to those of patients with blindsight. For example, on one occasion she said: ‘However, I do not understand that! You put something here. I do not feel anything and yet I go there with my finger. How does that happen?’. The anatomical description of the lesion was based only on CT images, but certainly, the primary sensory cortex (S1) had been severely affected by the stroke. Therefore, although the possibility of residual islands of tissue could not be completely discarded, the deep anesthesia developed after the lesion suggested complete damage to S1. Having only the lesional data available, it is difficult to say in this case which cortical or subcortical areas are responsible for processing without awareness. The authors pointed to the possibility that either a supplementary sensory area or subcortical structure could be responsible for the implicit processing of tactile signals. A similar dissociation between unawareness of the stimulus and the ability to indicate the stimulus location on the skin was also reported by Rossetti and colleagues [46]. They, however, found that their patient, although able to indicate the locus of the stimulation with a motor act, was not able to name the part of the body that was touched. Their conclusion was that their patient, in the absence of stimulus awareness, was able to indicate its position on the skin by relying on a system that encodes how a movement should be performed (how system) without constructing a more abstract representation of the location of the touch (where system). In their patient, structural brain damage was localized to the ventrolateral and ventroposterolateral nuclei of the thalamus. Functional studies on the same patient indicated hypometabolism not only in the damaged subcortical areas but also in the frontoparietal cortices, particularly S1, consistent with a thalamocortical diaschisis. In any case, we can conclude that the deafferentation of S1, due to the structural lesion, is again associated with the lack of phenomenal experience of touch. Regarding the implicit processing of the tactile stimulus, the authors proposed the somatic ipsilateral pathways as the substrate for the localization task, which potentially supports sensitivity in hemispherectomized patients. Crucially, a similar ‘blindtouch’ has been obtained in healthy subjects using a TMS procedure to affect the functioning of S1 by Ro and colleagues [47]. They found that despite transient loss of tactile awareness during TMS stimulation, subjects correctly localized the locus of stimulation although claiming not to feel the touches. All these data point to S1 as a necessary component of tactile awareness.

Another interesting model for the study of sensory awareness is that of the extinction phenomenon, where brain-damaged patients, although perfectly able to detect single stimulations, do not report the contralesional stimuli when presented simultaneously with ipsilesional ones. In other words, when two stimuli are simultaneously presented (double simultaneous stimulation, DSS), the one that should be processed by the lesioned hemisphere fades from awareness. For instance, in the visual domain, where most studies on extinction have been carried out, a stimulus presented on the contralesional visual field is not detected if another stimulus is presented in the ipsilesional visual field. This phenomenon is very frequent in right parietal patients, especially after a lesion in posterior parietal cortices (e.g., [48,49]). Many fMRI studies in these patients have shown that when left visual events are extinguished during DSS, activations of the right striate and extrastriate visual cortices are still present, although at a lower level with respect to the activations observed in single stimulation trials where stimuli are fully perceived (e.g., [48,50]). This may suggest that the residual processing might underlie some unconscious elaboration of extinguished contralesional visual stimuli. However, both behavioral and fMRI studies did not directly investigate whether the patients had implicit processing and what areas activate when subjects, although not seeing anything, gave correct responses regarding the unseen stimulus. We only know that a residual activation of V1 was observed in extinguished trials. Another important observation is related to the fact that in trials where extinction was not present, i.e., when patients were aware of both stimuli, an additional activation in V1 was observed, thus confirming its fundamental role in giving rise to conscious percepts. Interestingly, Driver and colleagues [49] also found that in conscious trials the prefrontal cortex was active, as in blindsight patients in the ‘aware’ mode. That unconscious visual processing can be present in patients with visual extinction was demonstrated years ago by Volpe and colleagues [51] for line drawing of objects presented either side of a fixation cross. They showed that patients could judge whether the two objects were the same or different despite the fact that they were not aware of the presence of the contralesional stimuli. Berti and coworkers [52] even demonstrated that correct same/different judgments were not limited to simple line drawings of two identical objects but extended to photographs of real different objects belonging to the same category and sharing the same name (e.g., a photograph of two different exemplars of a camera), thus showing a high level of analysis for unconscious visual events.

Extinction has also been studied, although more rarely, in the tactile domain (see Table 2 for comparison with visual extinction), where patients usually have lesions in the parietal cortex, as in visual extinction, but more caudally located in S2 (e.g., [53,54]). A seminal paper regarding the relation and anatomical correlates of tactile extinction and conscious touch perception is that of Sarri and colleagues [54]. They studied a patient with crossmodal tactile extinction in which a visual stimulus extinguished a tactile stimulus delivered on the contralesional side of the body. The patient presented an interesting pattern of awareness/unawareness responses with respect to the tactile stimulation because she showed crossmodal extinction of left touch on approximately half of DSS trials while being aware of left touch on the other half. Thus, it was possible to study the brain activation in the aware/unaware condition in the same patient. The authors found, similar to the visual extinction situations, that when the tactile stimulus in DSS was extinguished, there was still residual (but less than in the tactile stimulation-only conditions) activation of S1. In those DSS trials where the patient was instead aware of the tactile stimulus, there was additional activation of S1, together with similar activation of prefrontal areas as observed in previous studies in other sensory modalities. These results suggest that for a fully aware experience, not only the primary sensory cortices are needed; anterior areas related to the more abstract and general content of awareness are also needed (see discussion). It must be pointed out that in Sarri and colleagues’ study, implicit processing was not directly tested or observed, but only hypothesized based on the persistence of a certain level of parietal activation in the unaware condition. Implicit processing of extinguished stimuli has indeed been demonstrated in tactile extinction by Maravita [55], and Berti and coworkers [56] found that unconscious processing of somatosensory stimuli can reach the categorical level, as in visual extinction. Indeed, their patient was able to give same/different correct responses in DSS trials, when she had no idea what the object presented to the contralesional hand was, even when stimuli, although sharing the name, were completely different examples of the same object. Note that in this patient, extinction was always present on the contralesional hand even when it was positioned in the right space, suggesting that it was anchored to a body-centered frame of reference typical of the primary sensory areas. However, and somehow surprisingly, the detection of tactile stimuli presented to the healthy, ipsilesional, hand decreased when the good hand was positioned in the left space, indicating also an influence of some extrapersonal frame of reference in modulating tactile perception.

In summary, these findings showed that (a) there is the possibility of implicit processing of tactile information that does not reach awareness, as in the visual domain. We know that implicit processing in blindsight depends on the spared functioning of different brain regions, in particular the retino-collicular pathway. This does not exclude the possibility that in other domains, as in the tactile system, residual activations of primary cortices underlie other forms of unconscious perception (see Sarri and colleagues’ suggestion [54]). (b) Tactile sensation can be affected by the space where the limb receives the stimulation. Indeed, although in Berti and coworkers’ experiment, tactile extinction was consistently present also when the affected hand was moved in the good space, tactile perception of the unaffected hand placed in the contralesional space was somehow diminished, despite the fact that the sensory circuits of this hand, and in particular S1, were completely normal (not affected by any brain damage) [57]. (c) The normal functioning of the V1 and S1 are necessary but perhaps not sufficient for a fully aware experience. Indeed, S1 can be unaffected in extinction patients, and the patients may nonetheless be unaware of the tactile stimulus. We may infer that for a fully aware experience, other areas are involved, as suggested by the studies on blindsight in the visual domain (see discussion section). The involvement of the prefrontal cortex might play a crucial role in the emergence of sensory consciousness.

## 3. Illusory Experience of Touch in Healthy Subjects

### 3.1. Tactile Illusions

Illusion phenomena elicited in the tactile domain are one of the most fascinating examples of how our sense of touch can be deceived, leading to non-veridical tactile experiences (see Table 3). Of particular interest is the phenomenon of people who believe they have been touched when no actual tactile stimulation has been delivered. For example, in the ‘funneling illusion’ [58], two tactile stimuli applied on the skin are perceived on a single unstimulated skin site, localized at a central position with respect to the two stimulated areas. Chen and colleagues [59] investigated the brain correlates of the funneling illusion in the monkey brain, showing that the simultaneous stimulation of two fingers resulted in a single focal cortical activation located between the regions corresponding to the individual fingertip activations in the primary somatosensory cortex area 3b. This indicates that even in the absence of a tactile stimulus at the funneled site, the cortical response was comparable to that of an actual single-digit stimulation. Another example of this phenomenon is the ‘cutaneous rabbit illusion’ [60]. In this paradigm, a series of rapid taps are delivered sequentially along the skin of a participant, typically on the arm at two or more skin locations. After the tactile stimuli have been delivered, participants often perceive the sensation of a continuous hopping or ‘rabbit-like’ motion moving along the skin between the actual points of contact. Note that, for the illusion to occur, stimuli must be presented to adjacent regions of the skin (i.e., the illusion does not occur if stimuli are presented from the hand to the foot). This means that the cutaneous rabbit illusion might be constrained by the somatotopic organization of the somatosensory cortex [61], suggesting that this illusion is related to early stages of information processing [62]. The neural mechanisms behind this illusion have been investigated by Blankenburg and colleagues [63], who recorded the brain activity of participants during the perception of the illusion across the arm. What they found is that when participants experienced the illusion, there was significant activation in S1 at the location corresponding to the perceived but not physically stimulated intermediate points along the arm. This activation was also comparable in magnitude to that produced by actual tactile stimulation at those intermediate locations. Furthermore, researchers found activity in premotor and prefrontal regions, which may indicate an important role of these structures in the top-down modulation of somatosensory information processing in S1 during illusory (non-veridical) touch experiences. These striking data, which show that in healthy individuals it is possible to feel in a location that is different from the one that has been stimulated, again suggest a fundamental role of S1 in the generation of conscious awareness even when it is non-veridical.

### 3.2. Rubber Hand Illusion

Particularly interesting for understanding the process that gives rise to tactile awareness are the illusions in which tactile experience is modulated through multisensory stimulation that alters the way in which we perceive our body, as in the Rubber Hand Illusion (RHI) [64]. The RHI phenomenon is typically induced by having a participant place one hand out of sight (usually hidden behind a screen) while a fake rubber hand is placed in view, positioned in a congruent location with respect to the participant’s body. The experimenter then synchronously strokes both the hidden real hand and the visible fake rubber hand with brushes. After a short period of synchronous stimulation, many participants begin to feel as if the rubber hand is part of their own body and report the sensation of touch (the one given on the own hidden hand) on the rubber hand, where they see the stimulation. This phenomenon can be measured subjectively, by asking participants to give a rating of how much they feel the rubber hand as if it were their own hand, or more objectively, by measuring the drift of the perceived position of the participant’s hand towards the rubber hand. During the initial period of stimulation, there is an obvious bottom-up multisensory conflict between touch and proprioception (which coincide) and vision (which is in contrast to the other two modalities). The conflict is solved by recalibrating proprioception to the position where the subject sees the touch of the rubber hand. This would lead to a change in body references and to the self-attribution of the rubber hand that is embodied in the mental representation of the participant’s own body. However, for the illusion to occur, some constraints must be met: the fake hand must resemble a normal hand in size and shape (e.g., an elongated stick does not work), the fake and real hands must be of the same identity (i.e., both right or left hands), and the fake hand must be positioned in near space internally with respect to the real hand and in egocentric perspective (the rubber hand in allocentric perspective does not elicit the illusion) [23,65]. These constraints are obviously related to an acquired body representation related to previous knowledge of the appearance of bodies, a sort of pre-existing body representation, which in a top-down manner limits what can be considered part of a body [23]. Therefore, both bottom-up and top-down processes are needed for the illusion to occur (see also [66]). Note that self-attribution of the fake hand as part of the own body leads to localization of touch on the rubber hand, causing the counterintuitive effects of feeling on a fake hand. Interestingly, some studies have indicated that the incorporation of the rubber hand corresponds at an implicit level to a momentary disownership of one’s real hand. For example, Zeller and colleagues [67] showed how the tactile acuity (i.e., the ability to discriminate and perceive tactile stimuli) of the hand subjected to the illusion is significantly reduced, as is the amplitude of the somatosensory potentials evoked by a tactile stimulus (see also [68] for similar results in the motor domain). Therefore, the rubber hand illusion suggests how the phenomenal experience of touch is not exclusively dependent on direct sensory input but can be the result of a complex integration between multisensory stimulation and the sense of body ownership.

Many studies tried to identify the areas and circuits involved in the self-attribution of the rubber hand. According to some authors [69], recalibration of proprioception to the position where tactile stimulation is seen involves activation of frontoparietal circuits that include the intraparietal cortex, the supplementary motor area, and the cerebellum. However, when the illusion is achieved and tactile and somatosensory perception needs to be integrated with vision, the area that becomes active is in the premotor cortex, where bimodal visuotactile neurons are ideal candidates for the integration of visual and somatosensory signals [70,71]. Interestingly, an fMRI study showed that in participants who non-veridically perceived their arm as longer when an elongated rubber hand was used, an expansion of the area dedicated to the tactile representation of the arm was observed in S1 [72].

Taken together, these findings suggest that vision may affect tactile perception in different ways. In the case of RHI, vision wins over proprioception during multisensory stimulation by dragging tactile awareness from one’s own limb to the rubber hand. Furthermore, the data on RHI indicate that the subjective tactile experience involves, beyond the activation of S1, higher-order processing regions involved in the construction of body ownership.

### 3.3. Mirror Box Illusion and Phantom Sensation in Healthy Individuals

The mirror box illusion (MBI) was initially developed by Ramachandran and colleagues as a therapeutic tool intended to treat phantom limb pain experienced by amputees (a condition in which amputee patients can perceive pain in a limb that is no longer there). In this illusion, a mirror is placed perpendicular to the trunk midline of the patient’s body (with one limb on each side of the mirror), creating the illusion that the reflected limb is actually the opposite missing limb. This visual illusion can lead to significant changes in the patient’s body perception, including relief from phantom limb pain and alterations in perceived tactile sensations [73]. The fundamental theory behind the MBI is that under certain circumstances, visual feedback that creates the illusion that a missing limb is intact and moving can alter the brain’s mechanisms involved in body perception [74]. In one study, Ramachandran and Rogers-Ramachandran [75] showed that in amputees, it was possible to induce not only kinesthetic sensations but even an intermanual referral of touch. They asked a patient with a right limb amputation to look into the mirror at the reflection of his left hand so that the reflection was superimposed on the felt position of the right phantom limb. When the experimenter stroked individual fingers on the left hand, the patient reported a touch sensation in the exact symmetric location on his right phantom limb. This finding suggests that the persistence of brain maps dedicated to tactile processing and body representation in amputees may underlie the illusion created by the mirror as in healthy individuals (see below), despite the absence of the corresponding limb.

In later studies, the MBI was used to investigate various aspects of visuo-tactile integration also in individuals with intact limbs. In these participants, the MBI induces, as in the amputees, the feeling that one of their own limbs reflected in the mirror is actually the other limb hidden behind the mirror, and it has been widely used to induce visual/proprioceptive conflicts. In one particular example, Ro and colleagues [76] asked participants to look at the reflection of their right hand being brushed by the experimenter. In such conditions, the visual information (i.e., the mirror reflection of their right hand being touched) made it seem like their left hand was being touched instead. After a short time, many participants reported the feeling of being touched on the left real hand, while the tactile information was only delivered on the right hand. These data first show how the possible “embodiment” of the reflected right hand as the left own hand can have an effect on tactile awareness. Moreover, after the MBI procedure, the authors applied near-threshold electrical pulses delivered to the middle finger of the hand that was not actually stimulated during the MBI. It must be noted that usually, near-threshold electrical stimulations increase sensitivity when a real stimulation has previously been delivered on the skin. Accordingly, they found that the stimuli delivered at threshold level on the real left hand that underwent the illusion during the MBI were perceived more easily following exposure to the mirror, as if it was really stimulated. Interestingly, inhibiting the activity of the posterior parietal lobule with a TMS procedure abolished the ‘facilitation’ effect. These data demonstrated that the illusion of being touched on one hand when in fact stimulation had been given on the other hand has effects on the sensory parameters of the hand that did not receive any tactile stimuli, showing also, in the domain of this experiment, that the phenomenal non-veridical experience of touch has consequences for veridical touch perception. Furthermore, the fact that TMS on parietal areas inhibits the facilitation induced by the MBI shows that not only are these areas involved in touch modulation but that this effect is also verifiable for non-veridical tactile experiences.

The possible mechanisms behind the MBI are mainly ascribed to the mismatch between vision, touch, and proprioception [64,77]. As in RHI, the MBI reveals the brain’s tendency to rely more heavily on visual information when there are conflicting sensory inputs [78]. Different studies explored the neural correlates of the illusory touch in the MBI. Interestingly it was found (e.g., [79]) that stimulating the right hand (reflected in the mirror) created a medial shift in the cortical representation of that hand, which is closer to the cortical representation of the left hand, showing how S1 topography can adjust to accommodate for the “embodied limb”. These data show how tactile awareness is modulated by multisensory stimulations possibly integrated at the cortical level but is also modulated by subjects’ perception (veridical or not) of their own bodies. Moreover, the shift of the stimulated hand representation to the non-stimulated hand in S1 indicates that a change in tactile awareness is accompanied by altered activity in the primary sensory cortices.

### 3.4. Synchiric Errors

Recently, we reported a new phenomenon in the domain of illusory tactile experiences in healthy subjects using the MBI paradigm [80]. We delivered symmetrical and asymmetrical stimulations on the dorsum of the hands of participants while their left hand was out of sight (hidden behind the mirror) and the right hand was reflected in the mirror so that it resembled the own left hand. In this setup, during asymmetrical simultaneous stimulation of the two hands, participants not only saw the right hand reflected in the mirror as if it was the left hand but also saw it being touched by the stimuli given to the real right hand. This implied that when the right hand was touched in one position and the hidden left hand received the touch on a different position, subjects saw in the hand reflected in the mirror a touch that was ‘symmetrical’ to that given on the real right hand. What we found is that during asymmetrical stimulations, participants erroneously reported the touch on the left hidden hand in a position that was symmetrical to the stimulation on the right hand. We called this phenomenon “synchiric error” because it resembles synchiria [81,82], a phenomenon in which patients describe the feeling of being touched on two symmetrical sides of the body when only one side is stimulated. We ascribed this phenomenon to a combination of both bottom-up and top-down processes. The conflicting multisensory integration induced by our setup, that is, the feeling of touch on the left hand while seeing the touch on the right reflected hand (bottom-up sensory processing) together with a possible embodiment of the reflected right hand as the own left hand (top-down modulation of body ownership) might have induced the non-veridical sensory experience on the left hidden hand [80], in accordance with previous findings by Ro and colleagues [76].

## 4. Phantom Touch in Brain-Damaged Patients

### 4.1. Phantom Touch in the Absence of Body Illusion in Synchiric Extinction

Tactile perception in post-stroke patients is typically assessed by administering tactile stimuli to the patients’ hands, either unilaterally or bilaterally, and asking them to say yes when they feel the touch. On some occasions, they are also asked to indicate the locus of stimulation. Note that usually, in the clinical testing of tactile perception, bilateral stimuli are delivered in symmetrical positions on the two hands. This procedure helps to evaluate errors in reporting contralesional single stimuli, as in primary somatosensory deficits or neglect [83], but also the inability to detect contralesional touches during simultaneous bilateral stimulation, as in tactile extinction [84]. Additionally, this procedure is also used to identify phenomena such as synchiria [82], where patients experience bilateral tactile sensations from unilateral stimulation, and allochiria, where patients mislocalize contralesional stimuli to the ipsilesional side of the body [85]. However, we found that this standard procedure does not capture extinction errors when the patient who may present extinction with double symmetric stimulation still reports two stimuli because the perception of the stimulus on the healthy side induces a phantom feeling of touch on the affected side (synchiric errors). In other words, since the stimuli are given symmetrically, the synchiric error may be masked. We discovered this phenomenon in a study where stroke patients who apparently did not show any sign of tactile deficits under standard evaluation of touch showed extinction on the contralesional side using a new procedure called Tactile Quadrant Stimulation protocol (TQS) [86]. Following this procedure, we delivered tactile stimuli on one of the four quadrants of the patient’s hands. Single stimulations, symmetrical double stimulations, and in the crucial condition, asymmetrical double stimulation (ADS) of the hands were used. Patients had to report the side and position of the stimuli. Results showed that more than 50% of the patients, although correctly reporting a bilateral tactile sensation in ADS trials, indicated, as the locus of stimulation, symmetrical positions on the two hands. In other words, for the contralesional hand, they claimed to feel touch on the quadrant (that was not actually stimulated) that corresponded to the one stimulated on the ipsilesional hand. We called this non-veridical tactile experience ‘synchiric extinction’ because it resembles synchiria but selectively arose after a possible extinction of the actual stimulus during ADS trials (see Table 4). The synchiric extinction phenomenon may reflect neuroplastic mechanisms triggered by the brain lesion that unmask pathological bilateral touch representation following unilateral stimulation [87,88,89,90,91], which are normally inhibited in the healthy brain [81]. In synchiric extinction, the phantom touch is not related to the body ownership illusion but to the mechanisms of visual extinction that impact conscious processing of the tactile stimulus.

### 4.2. Phantom Touch and Body Illusion in Brain-Damaged Patients

In the next section, we shall discuss neuropsychological syndromes characterized by abnormalities in body perception and in the sense of body ownership (see Table 4), where tactile awareness is impaired or affected in different ways. As pointed out in the introduction, we can define the sense of body ownership as the conscious feeling of property over one’s own body with the consequence that subjective sensations are private states not shareable with others (see [92]). Moreover, we also have a feeling of continuity in time with our body, so that we recognize it as our own despite the passing of time [93].

A special point of interest in this definition for the discussion of touch is that body ownership makes body sensations unique to one’s self. This implies the exclusivity of the relationship between the perception of an event on one’s body and the property of that body. We have already seen how with particular procedures, it is possible to undermine this relationship in healthy subjects. We shall see even more counterintuitive behaviors in brain-damaged patients in the next paragraphs. Regarding perceptions and representations of the body, in the classical literature, a distinction is usually made between the concepts of body image and of body schema. Body image refers to conscious representations about visual features of the body and of its appearance, whereas body schema is a representation that unconsciously guides postural and motor processes in both action execution and perception of the body in space [21,26,94,95,96]. Although there is now a tendency to use more complex definitions of the various high-order components on which body ownership depends, we will still refer in some parts of our review to this distinction, considering it efficacious in capturing some phenomena observed in both healthy subjects and patients with brain injury.

### 4.3. Delusion of Disownership

One of the most surprising behaviors that can be observed after a focal brain injury is the explicit denial that a limb belongs to one’s own body. This pathological condition often accompanied by the attribution of the limb to another person is called somatoparaphrenia (SPP) [97] and is interpreted as a sort of discorporation (disownership). A causal role for SPP has been attributed to a complete alteration of somatic sensitivities. Indeed, the association between somatosensory deficits and the denial of belonging is very frequent. In many cases of SPP, an apparent total disturbance of sensation and particularly of touch seems to afflict the patient. However, Moro and colleagues [98] found in two SPP patients that when the contralesional arm (the one affected by the disownership) was moved to the space unaffected by the lesion, the patients were able to report tactile stimulations, although SPP did continue to persist. Therefore, this observation not only shows that SPP is not caused by sensory deficits but indicates that tactile awareness is modulated by the space in which the patient’s arm is [98]. Interestingly, when the unaffected arm was moved into the contralesional affected space, some tactile stimuli were not perceived (see also [56]). Similarly and even more striking is the behavior of patient FB, as described by Bottini and colleagues [99]. The patient, who after a right hemisphere stroke developed a complete paralysis contralateral to the brain damage, did not report upon routine neurological exam any tactile stimuli on her left arm. When the examiners brought the patient’s attention to the affected hand, the patient claimed that that hand was not hers and that it belonged to her niece, showing severe and persistent somatoparaphrenic behavior. At this point, the patient was told that she would receive a touch either on her right or left hand or on her niece’s hand. In the latter case, the examiners would again touch the left hand of the patient. The patient was asked to keep her eyes closed and to say if and where she had been touched. Surprisingly, the tactile anesthesia of the left hand, which was very consistent when the examiners were telling the patient that they were touching her hand (the one that was discorporated) disappeared (i.e., the patient reported tactile stimuli correctly) when the examiners said they were touching the hand of the niece. Considering that the patient was performing the task with her eyes closed, the only factor influencing her responses was the belief (true or false) about the hand ownership, induced by the examiners’ information. It is clear that in patient FB, the cortical areas deputed to the analysis of sensory stimuli had to be intact, given the patient’s ability to correctly report the touch on her left hand when she believed it was her niece’s hand being touched. As in Moro and colleagues’ study, the aware experience of touch depended upon the beliefs related to the hand ownership that counterintuitively led the patient to feel stimuli on someone else’s hand.

Finding a relationship between the non-veridical phenomenal experience reported by patients and objective, quantifiable data would allow us to grasp the neurological, not confabulatory, reality of the disownership phenomenon. For example, the existence of a relationship between the rejection of one’s own hand and changes in some physiological indices would be fundamental to tie the delusion to the neural mechanisms that likely determine it. Many studies have shown that approaching the body with a potentially painful stimulus (such as a needle or syringe) results in an increase in skin conductance (skin conductance response, SCR). This is considered an ‘anticipatory’ response due to the existence of circuits dedicated to the detection of potentially “threatening” stimuli approaching the body. These circuits would include the neural systems responsible for processing tactile and visual stimuli, signaling in advance possible harmful impacts on the body surface [70,71]. For the approaching stimulus to be considered dangerous, the subjects undergoing the experiment must consider the threatened body part as belonging to themselves. Under this assumption, if a body part is no longer considered one’s own, then the threat should not result in an increase in SCR. Romano and coworkers [100] registered the SCR in five patients with SPP, during the presentation of “painful” stimuli approaching the right (healthy) hand or the left (affected by the illusion of discorporation) hand and found that while for the right hand, the SCR increased as expected (the right hand is recognized as own), for the left hand, no anticipation response was found. This result is extremely indicative of the fact that the hand that is discorporated in the SPP patient is not treated as their own hand by the neural systems in charge of the body’s defense.

Another condition of relative disownership is one in which subjects, with no apparent brain injury and in the absence of psychiatric disorders, would like to amputate a limb. In this dramatic condition, termed xenomelia by Hilti and colleagues [101] but better known by the term body integrity dysphoria (BID), the ‘patients’ claim that the physical appearance of their body (i.e., what their body really looks like) does not match the mental image they have of it. Some patients may say that they feel ‘over-complete’ (e.g., [102]) and claim that they would feel “normally” complete with three limbs, which suggests the presence of a distortion of the body image, somehow similar to that of SPP patients (for a discussion of these disownership conditions, see also [32,103]). Particularly interesting for what we are discussing here is that some patients report the feeling that tactile stimulation is attenuated on the limb they would like to amputate, and the resulting impression is that the touched limb does not belong to them [104]. These subjective feelings could be explained by the fact that patients, wanting to get rid of the limb, try to ignore it. As a consequence of decreased attention, stimulations will be processed less effectively by the nervous system. Alternatively, the attenuated sensations could be due to less (or absent) representation of that limb in the brain. In this case, although the neural pathways for primary processing of tactile sensations are intact (these patients have no obvious nerve pathway lesions), the lack of a mental image of the affected limb with which to integrate them would prevent the full experience of the stimulation. Romano and colleagues [105] conducted a study aimed at understanding whether there was a difference, in patients with BID, in SCR responses when the tactile stimulus (given with a small needle) actually touches the patients’ skin (real stimulation) versus when the stimulus does not touch it (threat). The authors found that the SCR is increased when the stimuli touched the skin of the unwanted limb, while it was reduced when the stimuli did not touch it, which is when it was a threat. Therefore, in patients with BID, the decreased anticipatory response for the unwanted limb is similar to the response observed in SPP patients for the “discorporated” limb. In contrast, the response to actual limb stimulation was increased (see also [106]). The apparent contradiction between the increased response to actual stimulation and a decreased response to the threat was attributed by the authors to the key role played by attention in modulating SCR. Romano and collaborators have proposed that attention toward the unwanted limb is actually decreased on the assumption that dislike towards the affected limb is such that it leads patients to ignore it as much as possible. The diminished response to threat is therefore due to the fact that one does not expect to be threatened by something that does not exist (or that one wish did not exist). However, precisely because in BID the unwanted limb is treated as if it was not part of the own body, when it is actually touched, there will be a kind of startled response that increases the SCR to the actual stimulation. In an attempt to find the neural base of BID, Hilti and colleagues [101] conducted a structural study on a group of BID patients compared to a group from a healthy population. They found that in BID, there was a reduction in cortical thickness and volume in the superior parietal lobule and a reduced cortical surface area of the inferior parietal lobule, of a small portion of the frontal lobe, and of the primary somatosensory areas and the cortex of the insula. More recently Saetta and coworkers [107] also showed that the S1 area, related to the to-be-removed limb, and the right superior parietal lobule were less functionally connected to the rest of the brain. Although these abnormalities are very limited and quantitatively minor with respect to the lesion observed in brain-damaged patients, they nonetheless indicate the involvement of brain regions responsible for body representation and tactile elaboration. Interestingly, the study by Hilti and colleagues found that brain areas involved in the structural abnormalities in BID patients partially overlap with the areas that are damaged in SPP patients [108,109]), suggesting, therefore, a close link between the negative attitude towards an unwanted limb and the lesional pattern in which patients overtly deny the limb’s ownership. It is worth noting that the brain abnormalities found in patients with BID may be the effect of underutilization, repeatedly documented in these patients, of the unwanted limb and not the cause of the disorder. In any case, both behavioral and anatomical similarities between BID and SPP suggest that the structural abnormalities identified in the brain of these patients involve an alteration of complex circuits responsible for the integration of sensory information with body representation. In both BID and SPP patients, the partial or complete sense of disownership, possibly due to a sort of ‘amputation’ of the body image (congenital in BID, acquired after the stroke in brain-damaged patients, see also [32]), affects the way in which tactile sensation is experienced.

### 4.4. Delusion of Ownership

We recently described a neuropsychological disorder that could be considered the opposite of SPP in which something similar to what happens in healthy subjects during the RHI occurs. Patients suffering from it incorporate limbs of other people (that, in this context, we call ‘alien’ limb) when these are presented in positions compatible with their own body. In accordance with what Tsakiris and Haggard [23] described for the RHI, for the embodiment to occur, the ‘alien’ limb must be in an egocentric position internal to the patient’s own limb and of the same identity. We have called this disorder ‘pathological embodiment’ (PE) [32,110]. Usually, these patients have a lesion in the right hemisphere, and therefore the affected part of the body is the left one, most often the left hand. In the typical assessment setting, where the patient with PE is seated with both hands resting on the table, the examiner, standing behind the patient, places his or her left arm between the patient’s real left arm and trunk. At this point, there are three arms on the table, and the patient, without any multisensory stimulation maneuvers such as the one used to induce the rubber hand illusion, immediately recognizes as his/her own hand both the right own hand and examiner’s left hand. This does not happen when an alien right hand is positioned on the right (healthy) side where the patients do not have any ownership illusion. Note that this phenomenon is observed without the patients’ explicit refusal of the left own limb when the alien limb is not present. On the other hand, when the alien limb is present and is incorporated, only then do the patients deny that the limb affected by the lesion is their own, coherent with the general knowledge that a person cannot have three limbs (if an alien limb is incorporated, then one must be discorporated). Another important constraint of this illusion of ownership is that the ‘alien’ limb must be a real one, as PE does not occur with fake hands. Of course, the fundamental difference between RHI and PE is that while healthy individuals undergoing RHI know that the rubber hand is not theirs, patients with PE are convinced that the alien hand belongs to their body. As Frederique de Vignemont pointed out, ‘something can be considered ‘incorporated’ if it is treated as a part of the own body’ [21]. In accordance with this hypothesis, we have shown in several studies that the examiner’s alien hand is indeed treated like the patient’s own hand in several domains [111,112]. Here, it is important to mention how tactile awareness is modulated in PE patients. In one study, we asked patients to give a score relative to the perceived strength of stimulation on both hands (using a pinprick) [113]. The score ranged from 0 (‘I feel nothing) to 5 (‘I feel perfectly’). Patients were selected on the basis of having relatively intact touch sensation even on the contralesional hand, which thus acted as a control for the alien hand. We found that patients gave the same (high) scores when their own (healthy) right hand and alien left hand were stimulated under the conditions of embodiment described above. They also gave scores consistent with good touch perception on their own left hand when assessed in the absence of the alien hand. When the examiner’s right hand, which corresponded to the patient’s healthy hand, was stimulated, they felt absolutely nothing. Coherently with the subjective report of feeling on the alien hand, we found using SCR that the anticipatory response was identical and increased with respect to the absence of stimulation when the pinprick stimulation was presented on the own right hand and on the left alien hand, definitively showing that when the alien hand is incorporated, it is treated as the patient’s own hand [114]. Somehow unexpected was the fact that when the stimulation was presented to the left hand in the absence of the corresponding alien hand and therefore explicitly recognized by the patients as their own, no increase in the SCR was observed (as in SPP patients). We interpreted this result as indicating an implicit state of limb disownership for the contralesional hand, which in the presence of the alien hand is indeed not recognized as one’s own. Thus, in the face of correct explicit ownership over the contralesional hand when alien hands are not present, the implicit SCRs suggest unconscious disownership. This could explain the tendency of these patients to incorporate alien hands when conditions for incorporation occur. In PE patients, the brain injury has not completely damaged body representation because in the absence of alien hands, their behavior indicates good perception of body experiences at the explicit level. The lesional data, although showing damages of different cortical areas, did not show any specific cortex involved in the PE group compared the control group of brain-damaged patients without PE [110]. Instead, in the PE group, subcortical structures such as the corona radiate, and the superior longitudinal fasciculus seem to be specifically damaged. Interestingly, these structures are also lesioned in SPP patients, thus suggesting a basic similarity between the two phenomena. This pattern of lesions implies a possible disconnection between the different brain areas devoted to the body representation, thus impairing a full coherent sense of body ownership. The results would not be a complete disruption of body image but instead an unstable and fragile body representation that, as mentioned, makes patients willing to accept as their own any limb that is in a compatible and congruent position for being part of their body. Going back to the veridical and non-veridical experience of touch in PE patients, these data show that the phenomenal experience of touch (still possible because these patients do not have damage to S1) is modulated by the feeling of ownership over the own and alien hands, whether veridical or illusory.

## 5. Discussion

The experience that we have of ourselves is characterized under normal conditions by an unquestionable feeling of unity and coherence. Therefore, in everyday life, it is difficult to separate a single stream of consciousness from the whole of our conscious experiences. However, it is possible using specific experimental conditions together with the study of anomalous illusory situations to isolate one sensory experience from the others in order to study how conscious experience is constructed. In this review, we set out to study how evidence in both healthy subjects and brain-damaged patients can provide useful insights into a specific domain of awareness, which is tactile awareness.

### 5.1. S1 Is Necessary but Not Sufficient for Tactile Awareness

First, in the tactile domain, we can also observe implicit processing of information in the absence of any sensory experience. We have seen that this can happen both in the complete absence of the main pathways for primary sensory processes (as in blindtouch, where S1 is surely affected, like V1 in blindsight patients) and in conditions in which the primary sensory cortex is spared (like in tactile extinction or in visual extinction, where V1 and S1 are not affected). These observations suggest that both in vision and touch, primary sensory cues are necessary for tactile awareness (if they are destroyed, as in blindtouch and blindsight, no awareness of the processed stimuli is still possible). It must be noted that the fact that S1 is intact in extinction does not mean that S1 is not involved in awareness. Indeed, patients are completely aware of stimuli when they are delivered in isolation, either to the right or to the left limb. Unawareness in extinction occurs when the system is loaded with double simultaneous touches. In this case, the analysis of the touch that reaches the healthy and efficient cortex is facilitated (and then consciously reported) with respect to the one that would reach the lesioned hemisphere (which is neglected). Indeed, extinction can be considered a mild form of inattention or lack of spatial awareness due to lesions to the posterior parietal lobe (see [115] for a similar explanation of unilateral neglect). Therefore, the unawareness of touch with spared primary somatosensory cortex, observed in extinction, suggests that S1, although necessary, is not sufficient to generate a conscious tactile experience. Other higher-order brain areas must contribute to the emergence of a conscious experience of touch (see below). Returning to the role played by S1 in the processing of tactile stimuli, other data discussed in this review indicate its fundamental involvement in the emergence of awareness. For example, in tactile illusions, the perception of a stimulus referred to a point on the skin other than those actually stimulated (as in the funneling illusion) is accompanied by plastic changes in neural response localized in the primary sensory area. In other words, the non-veridical tactile awareness that emerges, although not related to the activation of the skin receptors actually stimulated by touch, nevertheless depends on the activation of S1. Similarly, in the cutaneous rabbit illusion, Blankenburg and colleagues [63] found an activation of S1 when subjects perceived the illusion at the location corresponding to the perceived but not physically stimulated intermediate points along the arm. Even more compelling is the evidence that the magnitude of activation observed during the illusion was comparable to that observed when the stimulus was effectively delivered at the intermediate location. Similarly, in the MBI, when the tactile stimulation of the right reflected hand in the mirror causes patients to feel the reflected hand as their own left hand, Egsgaard and colleagues [79] showed a medial shift of the right-hand representation in S1 toward the real hand that, although not really stimulated, feels the touch. Again, tactile awareness, even in a non-veridical condition, is accompanied by activation of S1. Finally, a finding that incontrovertibly points to the direct involvement of S1 in tactile consciousness is that inhibition of S1 with TMS results in a momentary blindtouch-like condition in healthy subjects [47].

S1 is certainly not the only area involved in conscious tactile experience. Avanzini et al. [116] proposed that tonic response to mechanical stimulation in the parietal operculum (SII) and the insular region may have a fundamental role in the emergence of tactile awareness. Sensory information is, indeed, transferred from S1 to S2, a parietal area that is connected to various circuits that influence the processing of S1 in a top-down manner [7]. The lesions that produce extinction are adjacent and sometimes correspond to these areas (see [53]), and we have just seen that in the presence of an intact S1 but with these parietal areas damaged, tactile awareness is suppressed. All these data point to the fact that the circuits involved in tactile consciousness are the same that are responsible for the processing of tactile information. That is consciousness seems not to be related to some superimposed amodal process that is common to all modalities. We proposed this view for motor awareness [7,117], where the area most damaged in anosognosia for hemiplegia (which is a disorder of awareness related to motor function) is the premotor area 6. However, in blindsight patients, the (amodal) prefrontal area 46 has been found to be active in both the fully aware mode and in the attenuated awareness in type 2 blindsight [43]. Interestingly, another area that we found to be selectively damaged in anosognosic patients is area 46. So, while the emergence of a conscious sensory experience certainly requires the activities of the primary processing areas, other amodal areas could contribute to conscious processing. For tactile processing, there are no studies that specifically evaluate the role of area 46 in tactile awareness. The picture that emerges, however, from studies conducted in other specific domains seems to suggest that different brain circuits are necessary but not sufficient to determine conscious experience and that amodal areas such as area 46 may also contribute to these circuits. A possible hypothesis is that the reciprocal connections between primary and amodal cortices guarantee the primary area to be a sufficient activation level for the emergence of awareness. This can be reached by back-projections from higher-order areas, including area 46, to S1 and S2. As Gallace and Spence pointed out, ‘We believe that a certain threshold level of activation in the circuit needs to be reached […] in order for awareness to be generated’ [7]. As blindsight studies have shown, consciousness might not be a matter of threshold activation but more of activity in specific circuits. However, the two hypotheses are not in contrast with each other, and it is possible to assume that even within a dedicated circuit, a certain threshold of activation is necessary to trigger the conscious experience.

### 5.2. Neural Bases of Processing without Awareness

If the lack of tactile awareness in blindtouch is ascribed to the damage to S1, less clear is the neural basis of processing without awareness, which is nevertheless present. One possibility is that similar to blindsight there is a separate, dedicated circuit that also supports non-conscious responses in the tactile domain. In this regard, Gallace and Spence [7] suggest in their model pathways from the thalamus to the posterior parietal cortex and to the premotor cortex that may sustain implicit processing. However, in their model, these pathways seem not to be segregated, as the retino-colliculo-extrastriate pathway in blindsight is. In particular, they suggested a route that goes from the posterolateral thalamic nuclei to the many different secondary and amodal cortices that are actually involved in many different spatial and cognitive processes. However, another interesting possibility has been proposed by Rossetti and colleagues [46]. In their patient with damage to the left ventrolateral and ventroposterior lateral nuclei of the thalamus, functional investigation also demonstrated important hypometabolism of the whole parietal cortex. Therefore, alternative thalamocortical pathways cannot be considered in this case responsible for processing without awareness. Interestingly, Rossetti and colleagues proposed that unaware processing could be carried out by ipsilateral somatic pathways. This would imply a bilateral representation of touch, as many authors have already proposed even if in other clinical disturbances. One possibility is that the ipsilateral representation is not as strong and efficient as the contralesional one, causing an activation in the ipsilateral pathways that is sufficient for implicit processing but not for explicit awareness. Note that Sarri and colleagues [54] have proposed for extinction that the survival of primary sensory cortex may sustain the implicit processing. However, in their study, they did not test the presence of implicit processing; therefore, no conclusion can be drawn in this respect. As for the other studies on sensory extinction with spared implicit processing, the hypothesis is that the brain damage affects spatial awareness, while leaving the pathways for processing the stimuli intact or still working (but at a lower level of efficacy). In the absence of awareness of space, awareness of touch or haptic recognition of objects is also prevented, although it is not directly affected by brain damage (see [115]). Further research would clarify the alternative pathways involved in non-conscious processing.

### 5.3. Space and Touch

Some data presented in this review clearly indicate how the conscious processing of touch is linked both to somatotopic representations in S1, being therefore anchored to the limb that has been stimulated and to spatial reference systems linked to extrapersonal space representations encoded by the posteroparietal areas [56,98]. Indeed, conscious reporting of tactile stimuli not only depends on the normal functioning of the neurons in S1 but also on the space where the limb (the body) receives the stimulations. Research on tactile awareness has demonstrated that space is a necessary pre-requisite for any other conscious experience. Therefore, a model of tactile awareness should take into account the possible reciprocal connections of S1 and S2 with the areas that subserve spatial representation. This is represented in the model of Gallace and Spence [7]. In their model, some of the circuits related to high-order processes that modulate tactile processing are represented in a unique block of functions containing the various frames of reference in which space is computed. Dedicated studies in the future should take into account the possibility of double dissociations between tactile awareness and specific spatial functions in order to better specify the complex relation between skin, body and space (see for instance, [25]).

### 5.4. Vision and Touch

Many of the studies presented in this review unequivocally indicate the modulatory effect of vision on touch when, under experimental conditions, a conflict between what the subject sees and what the subject feels is generated. It must be noted that many studies in the literature have shown the so-called ‘visual enhancement of touch’ (VET) [118], which is an improved tactile acuity when a subject sees a part of the body being touched. The VET could be due to a direct connection of the visual pathways with the areas of the sensory homunculus that in S1 somatotopically represent the part of the limb that is touched. Interestingly, Ro and colleagues [76] found, in the context of MBI illusion, that stimuli delivered at threshold level on the real left hand (the hand that underwent the illusion) were perceived more easily following exposure to the mirror, as if a VET occurred. However, the counterintuitive observation in this particular experimental setting is that VET would occur on a hand that was not stimulated. Indeed, the part of the body where the stimulus was delivered (the right hand reflected in the mirror that created the illusion that the left hand was touched) did not correspond to the part of the body where the enhancement in the post-illusion occurred (the illusioned left hand). Thus, despite the sure effect of vision on touch in MBI, this effect is entrained by the altered body representation induced by the illusion. Importantly, the fact that vision has an effect on touch does not mean that visual consciousness dominates over tactile consciousness [26]. For instance, the experiments discussed in this review on RHI and MBI show that although vision entrains touch, even vision per se is deceived because it basically does not distinguish between a rubber hand (as in RHI) or a mirror image of the hand (as in MBI) and a real hand. Therefore, vision affects touch but via body image. In a certain sense, it is the body image that guides touch perception even when it is damaged by a brain injury (as in the various ownership disorders we have discussed) or altered by incongruent multisensory stimulation (as in RHI and MBI). This implies that vision has access to body representation and, in particular, to the body image that consciously represents the visual features of our bodies.

### 5.5. Body Representation and Touch

Leaving aside the effects on touch of illusions like MBI and RHI in healthy individuals that we already discussed above, one of the most remarkable pieces of evidence that touch depends on body perception is the false beliefs about tactile awareness observed in patients with body ownership problems. In this review, we took into consideration both illusions of disownership, (as in somatoparaphrenia, SPP, and body integrity dysphoria, BID) and illusions of ownership (as in pathological embodiment, PE). As we have seen, in discorporation illusions, the affected limb is not only misrecognized verbally but also treated by body representation systems as not belonging to the patient’s body. This is shown incredibly convincingly when SPP patients with intact S1 (i.e., those who can feel touch on the discorporated hand) seem to lack tactile awareness on the discorporated hand (e.g., patient FB in [99]). Similarly, in BID, although the brain alteration is less pronounced than in SPP patients, tactile sensation is decreased. Consistent with the patient’s verbally re-ported conscious experience, the discorporated limb is treated by the body’s alertness and defense systems as an alien limb not belonging to the own body (see results in SCR). On the other hand, and somehow more strikingly, PE patients report a tactile sensory experience on the alien embodied hand. Again, the SCR is consistent with the non-veridical sensitivity reported by the patients, showing that in these cases the alert and defense systems treat and recognize the alien hand as belonging to the patient’s body. Surely these data again indicate that vision entrains tactile perception, because in both illusions in healthy subjects and in patients’ non-veridical tactile experiences, it is the vision of real and alien limbs being stimulated that produces the experience of touch. However, as already mentioned, the crucial difference between the two is that patients are convinced that a limb of their body does not belong to them (SPP and BID) or that an alien limb is part of their body. These beliefs are cognitively impenetrable and thus dependent on consistent alterations in body maps. We have already mentioned the distinction between body image and body schema. If we maintain this distinction, we may say that both are affected by the alteration of body representation. On one hand, to accept or refuse limbs must depend on altered body image, which is an impairment of how we see the body. However, the body schema is also affected because, for instance, we have demonstrated that in PE, false beliefs about own body affect the motor parameter in the execution of action with the unaffected hand [111]. Recently, we proposed a model of body ownership that, building on some suggestions in previous proposal [23], tries to explain the different observations in brain-injured patients. In particular, we pointed to the possibility that in both disownership and PE, damage to the way in which the body is conceived can determine patients’ behavior. Tsakiris and Haggard [23] proposed that the way in which we experience our body depends upon the match between bottom-up sensory information and a pre-existing body representation (PEBR) that is related to the knowledge about how bodies should be. This high-order representation might be altered in patients with body ownership disorders. However, Tsakiris [92] did not distinguish between general knowledge about bodies and more specific knowledge about one’s own body. So, we propose that there must be at least two different representations that can be linked to body image. A pre-existing body representation related to general knowledge about bodies (PEBR-G) and a pre-existing body representation related to knowledge about one’s own body, which we call pre-existing body representation-own (PEBR-O). These representations can be considered priors that predict how bodies should appear, whether they are our own bodies or those of others. In the patients described in this review, PEBR-G is intact. Indeed, patients know how human bodies are structured and that body parts are made of biological matter. This knowledge constrains not only the RHI in healthy subjects but also the embodiment of alien limbs in PE patients (alien limbs are not always incorporated, but only when they are real hands and in a position compatible with the patient’s body). What is impaired in patients with body ownership disorder is the PEBR-O. Whether rejecting one’s own limb or accepting someone else’s limb as one’s own, patients can have a disturbance in recognition of their own limbs. Bottom-up multisensory stimulation that should ensure veridical processing of one’s own limbs and the limbs of others does not match the PEBR-O, which is affected by the lesion. In this latter case, we may say that the prior does not work properly and may not be able to form predictions about the own body. As a consequence, the property of the affected limb becomes uncertain, unstable, and susceptible to illusory processes. The distinction between general knowledge about bodies and specific knowledge about one’s own body must be considered when constructing models that take tactile consciousness into account. What is interesting for the present discussion is that when PEBR-O is damaged, it creates an illusion of ownership that pervades all bodily experience and affects sensory consciousnesses in a top-down manner, regardless of where stimulations are given on the body. The altered non-veridical higher-order representation of one’s body wins the somatotopic representation of touch generated by skin stimulation, demonstrating once again that the integrity of S1 is not a sufficient condition to generate a veridical experience of the event that impacts our body.

## 6. Conclusions

This review discussed how tactile consciousness can be generated from body stimulations even under illusion conditions in normal subjects and patients with brain damage and body ownership disorders. The different studies showed, albeit in different experimental situations, that it is possible to have an experience of touch even when one’s own body is not stimulated and, conversely, to deny having felt touch when neural conditions allow it. Overall, the studies suggest that consciousness of events is constructed within a sensory modality (touch in this review) in which primary sensory cortices interact with high-order brain areas devoted to space and body awareness. The neuropsychological syndromes and the research on normal subjects described in the review suggest the existence, both cognitively and neurobiologically, of a heterogeneous structure of conscious processes, as opposed to the idea of a unitary structure and indicate how consciousness is not a function superimposed hierarchically on other cognitive activities with a monolithic and in-separable structure; rather, it is a distributed property in the brain that is inextricably implemented in the circuits dedicated to the various cognitive, somatosensory, and motor functions.

Considering the focus of our review, namely the analysis of the construction of the conscious experience of touch, many topics, related to tactile processing were not covered by us. In particular, we did not cover the involvement of touch in cognitive (e.g., object recognition) and affective (e.g., the role of touch in interpersonal relationships) systems. In addition, regarding the relation between touch and body, there are many other situations of altered body representations that deserve to be considered and discussed in relation to tactile experiences, e.g., anorexia nervosa. It would also be interesting to report a comprehensive meta-analysis of brain lesion mapping in patients with body ownership disorders (e.g., SPP, BID, and PE); however, studies devoted to this topic are still scarce.

## Figures and Tables

**Table 1:** Similarities between Blindsight and Blindtouch.

	Blindsight	Blindtouch
Description	Ability to localize visual stimuli without phenomenal experience	Ability to localize tactile stimuli without phenomenal experience
Cause	Damage to primary visual cortex (V1)	Damage to primary somatosensory cortex (S1)
Brain Structures Involved in Unaware mode	Subcortical structure (Superior colliculus)	Subcortical structure (Thalamus *, supplementary sensory areas, Ipsilateral pathways *)
Brain Structures Involved in Aware mode	V1, prefrontal cortex (Area 46)	Primary somatosensory cortex (S1)

‘*’ Indicates that the structures reported in the studies are hypothesized, not directly documented.

**Table 2:** Similarities between visual and tactile extinction.

	Visual Extinction	Tactile Extinction
Description	Lack of awareness of contralesional visual stimuli during DSS	Lack of awareness of contralesional tactile stimuli during DSS
Cause	Posterior parietal cortex	Parietal cortex (SII)
Influence of Spatial Context	Affected by location of stimulus within visual field	Affected by location of the limb receiving stimulation

DSS = Double simultaneous stimulation.

**Table 3:** Summary of non-veridical tactile experiences in healthy participants.

Phenomenon	Testing Protocol	Key Finding	Neural Signature
Funneling Illusion	Simultaneous stimulation of two adjacent skin points	The two points stimulated are perceived at a single unstimulated site between the two stimuli	Single focal cortical activation in S1 localized between regions corresponding to individual skin sites stimulated
Cutaneous Rabbit Illusion	Sequence of taps delivered sequentially along the skin, typically on the arm	Sequential rapid taps are perceived as a continuous hopping motion along the skin between actual points of contact	Activation in S1 at perceived but unstimulated points along the arm
Rubber Hand Illusion	Synchronous stroking of hidden real hand and visible fake rubber hand	Feeling that the fake rubber hand is part of one’s body	Frontoparietal circuits, intraparietal cortex, supplementary motor area, cerebellum, premotor cortex, S1
Mirror Box Illusion	Mirror placed perpendicular to trunk midline, reflecting one limb while the other limb is hidden	Illusion that the reflected limb in the mirror is the opposite limb	S1, cingulate cortex, prefrontal cortex

**Table 4:** Summary of pathologies that can cause phantom touch.

Phenomenon	Description	Testing Protocol	Brain Areas Involved
Synchiric Extinction (SE)	A non-veridical tactile experience where patients feel touch on an unstimulated location in double stimulation trials	Tactile Quadrant Stimulation protocol (TQS)	Internal capsule, putamen and caudate nucleus
Somatoparaphrenia (SPP)	Patients deny ownership of a limb, often attributing it to another person	Questionnaire testing phenomenal experience	Mainly subcortical regions (thalamus, basal ganglia, amygdala, internal capsule, corona radiata and SLF) and insula
Body Integrity Dysphoria (BID)	Individuals feel over-complete and desire limb amputation	Questionnaire testing phenomenal experience	Superior parietal lobule, inferior parietal lobule, S1, insula
Pathological Embodiment (PE)	Patients claim that an alien limb belongs to them	Questionnaire testing phenomenal experience	Mainly subcortical regions (corona radiata, SLF)

SLF = Superior longitudinal fasciculus.
